# Bacterial ring rot of potato caused by *Clavibacter sepedonicus*: A successful example of defeating the enemy under international regulations

**DOI:** 10.1111/mpp.13191

**Published:** 2022-02-10

**Authors:** Ebrahim Osdaghi, Jan M. van der Wolf, Hamid Abachi, Xiang Li, Solke H. De Boer, Carol A. Ishimaru

**Affiliations:** ^1^ Department of Plant Protection College of Agriculture University of Tehran Karaj Iran; ^2^ Business Unit Biointeractions and Plant Health Wageningen University and Research Wageningen Netherlands; ^3^ Canadian Food Inspection Agency, Charlottetown Laboratory Charlottetown PE Canada; ^4^ Department of Plant Pathology University of Minnesota St Paul MN USA

**Keywords:** actinobacteria, coryneform bacteria, *Microbacteriaceae*, quarantine pathogen, Solanaceae, *Solanum tuberosum*

## Abstract

**Background:**

Bacterial ring rot of potato (*Solanum tuberosum*) caused by the gram‐positive coryneform bacterium *Clavibacter sepedonicus* is an important quarantine disease threatening the potato industry around the globe. Since its original description in 1906 in Germany, management of ring rot has been a major problem due to the seedborne nature (via seed tubers not true seeds) of the pathogen allowing the bacterium to be transmitted long distances via infected tubers.

**Disease symptoms:**

On growing potato plants: interveinal chlorosis on leaflets leading to necrotic areas and systemic wilt. On infected tubers: vascular tissues become yellowish brown with a cheesy texture due to bacterial colonization and decay.

**Host range:**

Potato is the main host of the pathogen, but natural infection also occurs on eggplant, tomato, and sugar beet.

**Taxonomic status of the pathogen:**

Class: *Actinobacteria*; Order: *Actinomycetales*; Family: *Microbacteriaceae*; Genus: *Clavibacter*; Species: *Clavibacter sepedonicus* (Spieckermann and Kotthoff 1914) Li et al. 2018.

**Synonyms (nonpreferred scientific names):**

*Aplanobacter sepedonicus*; *Bacterium sepedonicum*; *Corynebacterium sepedonicum*; *Corynebacterium michiganense* pv. *sepedonicum*; *Clavibacter michiganensis* subsp. *sepedonicus*.

**Microbiological properties:**

Gram‐positive, club‐shaped cells with creamy to yellowish‐cream colonies for which the optimal growth temperature is 20–23°C.

**Distribution:**

Asia (China, Japan, Kazakhstan, Nepal, North Korea, Pakistan, South Korea, Uzbekistan, the Asian part of Russia), Europe (Belarus, Bulgaria, Czech Republic, Estonia, Finland, Georgia, Germany, Greece, Hungary, Latvia, Lithuania, Norway, Poland, Romania, European part of Russia, Slovakia, Spain, Sweden, Turkey, Ukraine), and North America (Canada, Mexico, USA).

**Phytosanitary categorization:**

CORBSE: EPPO A2 list no. 51. EU; Annex designation I/A2.

## Graphical abstract

We provide an updated overview of biology and epidemiology, genomics features, and virulence determinants as well as management strategies of bacterial ring rot of potato caused by *Clavibacter sepedonicus*.

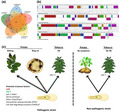

## 1 TAXONOMIC HISTORY OF THE PATHOGEN

In 1905 a previously unreported bacterial disease named “potato ring rot” was described in Germany (Appel, 1906) and the causal agent was named *Bacterium sepedonicum* (Spieckermann & Kotthoff, 1914), which was later changed to *Aplanobacter sepedonicus* describing the nonmotile rod‐shaped bacterium (Smith, 1920). Subsequently, gram‐positive plant‐pathogenic bacteria were transferred to the genus *Phytomonas*, and the potato ring rot pathogen was named *Phytomonas sepedonica* (Bergey et al., 1923). As the genus *Phytomonas* encompassed both gram‐negative, motile, green‐fluorescent (now known as *Pseudomonas* spp.) and gram‐positive, nonmotile, yellow/orange‐pigmented (now known as *Clavibacter* spp.) bacteria, the proposed reclassification was not accepted by most bacteriologists at that time. Thus, Dowson (1942) transferred the gram‐positive coryneform plant‐pathogenic bacteria into the genus *Corynebacterium* (“club” bacterium) (Lehmann & Neumann, 1896), and the potato ring rot pathogen was named *Corynebacterium sepedonicum*.

The name *Corynebacterium sepedonicum* was used for over 40 years until Davis and colleagues proposed the genus *Clavibacter* for gram‐positive coryneform plant pathogens containing 2,4‐diaminobutyric acid as a component of cell wall peptidoglycan (Davis et al., 1984). The ring rot pathogen was reclassified as a subspecies of the type species *C. michiganense* (*C. michiganense* was the original form of what is presently known as *C. michiganensis*). Although previously known as standalone species, the close similarity in biochemical and physiological characteristics was proposed to warrant subspecies classifications within *C*. *michiganense*. Host specificity was considered insufficient to justify differentiation at the species level. Hence, five members were classified as subspecies of the complex species *Clavibacter michiganense* and the ring rot pathogen was named as *Clavibacter michiganense* subsp. *sepedonicum* as one of the five subspecies within the species (Davis et al., 1984). Other species of *Clavibacter*, such as *C. xyli*, were later reclassified in different genera (Davis et al., 1984; Evtushenko et al., 2000). Until the late 1980s, the ring rot bacterium was called *C. michiganense* subsp. *sepedonicum* (Davis, 1986). However, under the nomenclature rules of bacterial taxonomy, in subsequent years the name was changed to *Clavibacter michiganensis* subsp. *sepedonicus* corrig. (Davis et al., 1984; Spieckermann & Kotthoff, 1914).

By the beginning of the genomic era, high‐throughput whole‐genome sequencing technologies initiated a substantial advancement in the understanding of population structure, phylogeny, and taxonomic relationships of phytopathogenic actinobacteria (Thapa et al., 2019). Several studies have highlighted the high genetic diversity and phylogenetic distances among members of *Clavibacter*. Hence, a reclassification of *Clavibacter* spp. into six new species was proposed based on genomic sequence comparisons, for example average nucleotide identity (ANI) and digital DNA‐DNA hybridization (dDDH) indices (Li et al., 2018). The original subspecies of *C. michiganensis* sensu lato were elevated to the species level, and the potato ring rot pathogen was reclassified as *C*. *sepedonicus*. Comparative genomic and phylogenetic analyses using publicly available genome sequences of the genus support the classification proposed by Li et al. (Osdaghi et al., 2020a). *C. sepedonicus* is a monophyletic taxon comprising only the strains originated from potato (Osdaghi et al., 2020a). Multilocus sequence analysis (MLSA) of concatenated *atpD*, *dnaK*, *gyrB*, *ppK*, *recA*, and *rpoB* gene sequences illustrates the current taxonomic position and phylogenetic relationships of *C. sepedonicus* among species of *Clavibacter* (Figure S1).

## 2 DISEASE SYMPTOMS

**FIGURE 1:**
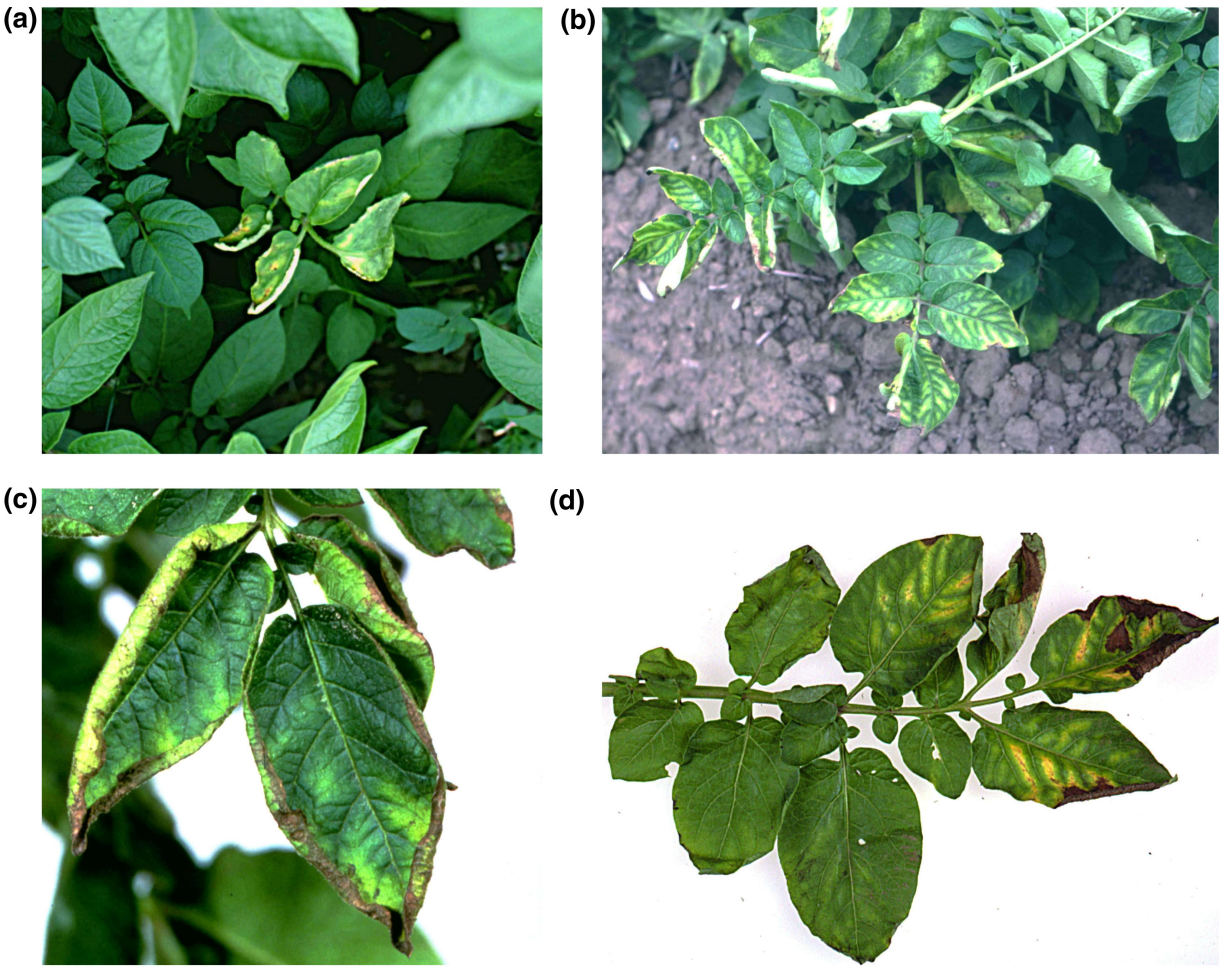
Field symptoms of bacterial ring rot caused by *Clavibacter sepedonicus* on aerial parts of potato plants. Interveinal spaces of the leaves become light green to pale yellow (a). Leaves then start to wilt and became slightly rolled at the margins (b). As the disease progresses, leaves become necrotic, starting from the margins (c). Infected leaves are often reduced in size and plants are occasionally stunted or even may eventually die (d)

Symptoms of ring rot disease are found frequently on potato plants during the growing season in the field and infection can remain latent for a prolonged period (Franc, 1999; Nelson, 1982). In the early stages of symptom development on growing potato plants, the interveinal spaces of the leaves become light green to pale yellow (Figure 1a,b). Leaves then start to wilt and became slightly rolled at the margins (Romanenko et al., 2002; Figure 1c). As the disease progresses, leaves become necrotic, starting from the margins (De Boer & Slack, 1984). Leaves and tubers are often reduced in size and plants are occasionally stunted. Infected leaflets, leaves, or even stems may eventually die (Figure 1d). Field symptoms vary from no detectable symptoms under low disease severity to complete necrosis of the leaves in cases of severe infections (Kawchuk et al., 1998). Under favourable environmental conditions, that is, cool and humid weather, overall wilt is observed and the entire plant can collapse.

**FIGURE 2:**
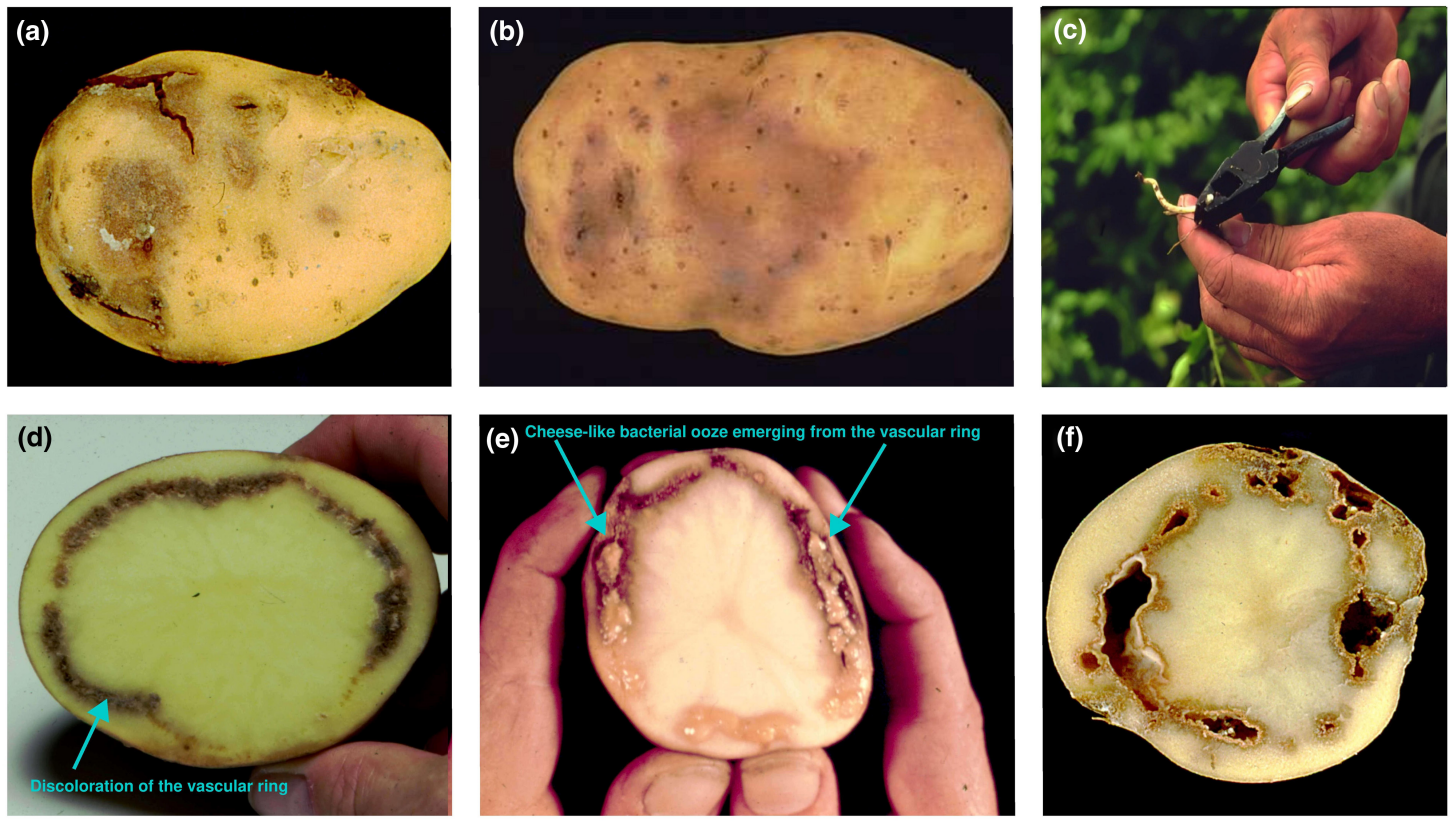
Symptoms of bacterial ring rot caused by *Clavibacter sepedonicus* on potato tubers. On intact tubers, surface cracks and dark blotches immediately beneath the periderm become visible (a). The surface of the severely infected tubers turns reddish‐brown (b). Milky exudate can sometimes be squeezed from wilted stems near the point of attachment to the tuber seedpiece (c). Ring rot symptoms can be observed by cutting the tuber longitudinally through the heel end where the tuber was attached to the stolon (d). When the tuber is gently squeezed, pillars of cheese‐like material emerge from the vessels (e). In the case of severe infection the two parts of the cortex may be separated and the entire tuber ultimately can rot (f)

Ring rot symptoms in tubers are usually observed after harvest and during storage (Gryń et al., 2020). Severity of tuber symptoms ranges from no detectable symptoms to complete breakdown of the vascular ring extending throughout the tuber (Kawchuk et al., 1998). As for the outer side of the infected tubers, surface cracks and dark blotches immediately beneath the periderm may become visible (Figure 2a,b). Ultimately the entire tuber can rot. Unlike the maceration caused by potato soft rots, bacterial ring rot is usually odourless. As described by Council Directive 93/85/EEC (EU, 1993), symptoms in tubers start as slight glassiness or translucence of the tissue without softening around the vascular system. Slight discolouration of the vascular ring at the heel end to a yellowish coloration is observed and when the tuber is gently squeezed, pillars of cheese‐like material emerge from the vessels (EFSA et al., 2019). Milky exudate can sometimes be squeezed from wilted stems near the point of attachment to the tuber and vascular tissues in these stems may appear brown (Howard et al., 1994; Figure 2c). On infected tubers, symptoms can be observed by cutting the tuber longitudinally through the heel end where the tuber was attached to the stolon (Figure 2d,e). Higher incidence of tubers with disease symptoms is found after a storage period. In seed tubers stored for several months—depending on the storage conditions—ring rot symptoms include vascular tissue discolouration becoming creamy yellow and soft cheesy in texture where milky droplets of bacterial slime are exuded when tubers are cut and squeezed. In the case of severe infection, the two parts of the cortex may be separated and the surface of the tuber turns reddish‐brown (Figure 2f).

Symptoms are rather variable and can easily be mistaken for other potato diseases such as brown rot of potato (*Ralstonia solanacearum*), potato late blight (*Phytophthora infestans*), potato wilt (*Verticillium albo‐atrum*), and stem canker (*Thanatephorus cucumeris*), or with senescence, drought or mechanical damage. As for brown rot of potato caused by *R*. *solanacearum*, symptomatic potato plants possess brown discolouration and necrosis of vascular tissues on the above‐ground sections. Furthermore, bacterial ooze exudation is frequently observed on the eyes and stolon attachment of potato tubers (Sedighian et al., 2020). Potato late blight caused by *P*. *infestans* on potato tubers includes wet and dry rots. Visual field inspection of potato plants as a standalone approach is not recommended for general surveillance. Secondary infections can mask typical ring rot symptoms.

## 3 ECONOMIC IMPACT OF THE DISEASE

Although in a regular production system the economic damage caused by direct crop loss is low, in the case of latent infections costs due to rejection of infected seed lots, as a control measure and by loss of export markets, are high. When seed tubers are injured by cutting or by using picker‐type planters, the infection percentage can be up to 80% (EFSA et al., 2019). In the European Union (EU) zone, ring rot outbreaks in seed potatoes are followed by eradication procedures, resulting in a relatively low disease incidence in seed. In the period from 2006 to 2015, annually 50,000–80,000 tests were conducted, from which only five to 100 tests were found to be positive. The incidence in ware potatoes (potatoes destined for human consumption in their original form), however, is still high. Between 25,000 and 33,000 samples were tested, from which 1000–2500 were positive (Baker et al., 2019). Based on expert judgments, the proportion of yield losses was estimated to be relatively low in seed (0.035%) but high (3.09%) in ware potato (EFSA et al., 2019). The time between introduction in potato and its detection was estimated to be 3.5 years, which means that for a long time the pathogen can spread unnoticed (EFSA et al., 2019). This is important in particular for farmer‐saved seed, which is still 70% in the EU. The quarantine status of the pathogen in the EU region was therefore maintained in a recent revision of the European and Mediterranean Plant Protection Organization (EPPO) A1 and A2 lists (Picard et al., 2017). The ring rot pathogen remains as a regulated plant pathogen in seed potato production in North America. In Canada, the disease was suppressed and functional eradication achieved in some regions largely due to the regulations enforced through the Canadian Seed Certification Program introduced in 1997. In the USA, seed certification is regulated at the state level with coordination of standard protocols as recommended by the Potato Association of America. Detection of the pathogen in seed potatoes results in loss of certification of all seed lots on a farm, requirements for disinfection of equipment and stores, and other domestic regulatory actions due to the zero‐tolerance policy for this disease. Another direct consequence is the rejection of seed potato in international trade. As the result of continuous efforts toward functional eradication, discovery of ring rot disease in the field is rare. In Canada, for instance, there has not been any report of observing the disease in the field during last 20 years despite routine scouting by the regulatory agency. In the USA, reports of ring rot disease in seed potato are similarly uncommon. With an effective certification programme for seed potato, the disease occurs only sporadically and generally at low levels in regions where it is endemic. However, it remains prevalent in countries where formal seed certification is absent.

## 4 HOST RANGE OF THE PATHOGEN

Potato (*Solanum tuberosum*) is the only economic host of *C*. *sepedonicus* (Figure S2a–c), while under laboratory conditions the bacterium is also capable of infecting other *Solanum* species such as tomato (*S. lycopersicum*), eggplant (*S. melongena*), and buffalo bur (*S. rostratum*) (van der Wolf et al., 2005). As for the nonsolanaceous plant species, the pathogen can induce disease symptoms on oilseed rape (*Brassica napus*), stinging nettle (*Urtica dioica*), and sugar beet (*Beta vulgaris*) plants (Bugbee et al., 1987; Ignatov et al., 2018; Pastrik et al., 2004; van der Wolf et al., 2005). On sugar beet, the pathogen causes severe wilt, infected young petioles are curled, and whole leaves are distorted (Figure S2d–f). Vascular tissues in the longitudinal root cut are brown to deep brown (Ignatov et al., 2018). Only eight out of 40 sugar beet cultivars of Russian and German origin were found susceptible to the ring rot pathogen. Recently, natural occurrence and pathogenicity of *C*. *sepedonicus* on tomato was reported by Van Vaerenbergh et al. (2016) in Belgium. Tomato plants infected by *C. sepedonicus* exhibited yellowing and necrosis of the leaf mesophyll, withering of leaflets, and wilting of whole leaves. In artificial inoculation of tomato plants using *C*. *sepedonicus* strains isolated from natural infection of tomato, the bacterium induced flaccidity and chlorosis of leaf margins, wilting or necrosis of individual leaf parts, and finally wilting of whole leaves. Conversely, Ignatov et al. (2019) isolated *C*. *michiganensis* strains from potato plants in Russia that exhibited chlorosis, leaf necrosis, and wilting of whole leaves and plants, while veins around potato tuber eyes were brown on cross‐sections. The *C. michiganensis* strains isolated from potato induced severe symptoms (interveinal chlorosis, mottling, and wilting) on both tomato and potato plants (Ignatov et al., 2019). Epiphytic growth on nonhost plant species is one of the survival methods of several coryneform plant‐pathogenic bacteria (Harveson et al., 2015; Osdaghi et al., 2018c), but has never been evidenced for *C. sepedonicus*. After stem inoculation, *C. sepedonicus* was successfully isolated from stem samples of maize, bush bean, broad bean, oilseed rape, and pea (van der Wolf et al., 2005). However, after root inoculations, the pathogen failed to survive in these crops. After inoculation of 12 solanaceous weeds, including the widely distributed *S. nigrum* and *S. dulcamara*, *C. sepedonicus* was only able to establish an infection in *S. rostratum* (van der Wolf et al., 2005). In fields naturally infested with ring rot‐diseased potato plants, the pathogen was detected in roots but not in stems of *Elymus repens* plants growing through rotten potato tubers, and in some *Viola arvensis* and *Stellaria media* plants (van der Wolf et al., 2005). The bacterium has been reported to cause disease in eggplant, tomato, and sugar beet. In particular, sugar beet, as a rotation crop of potato, may play a role in the epidemiology of the pathogen. Genomic features and evolutionary dating of *C. sepedonicus* suggest that there was recent adaptation for life in a restricted niche where nutrient diversity and competition are low, correlated with a reduced ability to exploit previously occupied complex niches outside the plant (Bentley et al., 2008; Bugbee & Gudmestad, 1988).

## 5 BACTERIOLOGICAL FEATURES

*C. sepedonicus* is a gram‐positive, rod‐shaped, short, and nonmotile bacterium (Hayward & Waterston, 1964). The pathogen is aerobic but slow growth can occur under anaerobic conditions. Its optimal growth temperature is 20–23°C (Davis et al., 1984). Gram‐stained cells may appear slightly club‐shaped and have a tendency to be in pairs in L‐ or V‐formation. Cells from fresh culture grown on laboratory media are sometimes quite pleomorphic, with cell morphologies ranging from large globose forms to the typical short, slightly club‐shaped rods (EFSA et al., 2019; Li et al., 2018). Plant‐pathogenic coryneform bacteria are well known for producing a variety of lipid‐soluble carotenoid pigments on culture media (Harveson, 2015; Osdaghi & Lak, 2015; Osdaghi et al., 2016) while *C*. *sepedonicus* along with the sugarcane ratoon stunting pathogen *Leifsonia xyli* subsp. *xyli* are the only two that characteristically do not produce pigments (Davis, 1986). Colonies of *C. sepedonicus* are creamy or white‐yellowish cream on general culture media (Davis, 1986). It has been hypothesized that insertion sequence (IS) elements may play a role in generating naturally occurring mucoid and nonmucoid variants of *C. sepedonicus* or the reported change from mucoid to nonmucoid morphology triggered by heat or nutrient stress (Bentley et al., 2008). Even using semiselective media (see below), nonmucoid phenotypes of the pathogen can escape detection due to a slower growth rate and lack of serologically recognizable epitopes on exopolysaccharides (Lewosz & Pastuszewska, 1995). To address this issue, petiole and stem injections of potato or eggplant can be used to enhance growth of low pathogen populations (Alivizatos, 1989; Bishop & Slack, 1987).

## 6 GENETIC DIVERSITY AND POPULATION STRUCTURE

Although several comprehensive investigations have so far been conducted to estimate the genetic diversity and population structure of other coryneform plant‐pathogenic bacteria, for example, *C. michiganensis* (Ansari et al., 2019; Jacques et al., 2012; Osdaghi et al., 2018a) and *Curtobacterium flaccumfaciens* (Osdaghi et al., 2018b), much less information is available about the worldwide population structure of *C. sepedonicus*. The ring rot pathogen is genetically, serologically, and biochemically relatively homogeneous. The pathogen displays a high level of homogeneity in carbohydrate utilization and in enzymatic activity (Palomo et al., 2006). The main variations among the strains are observed in their virulence/aggressiveness, the amount of extracellular polysaccharide (EPS) or bacterial slime produced on culture media. Rep‐PCR techniques, however, revealed no polymorphism and genetic diversity among fluidal and nonfluidal strains, indicating that relatively small genetic differences determine the fluidity (Fousek & Mráz, 2003). High genetic similarity was observed among *C. sepedonicus* strains by hierarchical analysis of *Hin*dIII and *Eco*RI genomic fingerprints where the clustering pattern was in congruence with disease severity on eggplant and potato, population size on potato, and ability to induce a hypersensitive response (HR) on tobacco (Brown et al., 2002a). Variable numbers of tandem repeat (VNTR) and PCR melting profiles based on the melting temperature analysis of *Bam*HI restriction fragments of chromosomal DNA were used to assess the intraspecies diversity of *C. sepedonicus* strains (Żaczek et al., 2019). The first complete genome sequence of *C*. *sepedonicus* (ATCC 33113^T^) became available in 2008 (Bentley et al., 2008). Its genome contains 106 copies of IS elements, which appear to have been active in extensive rearrangement of the chromosome compared to that of *C*. *michiganensis*. Of the four IS elements detected, the most prevalent element is IS*1121* with 68 copies encoded on the chromosome and two and one copies on the circular plasmid pCS1 and linear plasmid pCSL1, respectively. When a fragment of IS*1121* from pCS1 was used as a probe, restriction fragment length polymorphisms differentiated 10 strains of *C. sepedonicus* (Mogen et al., 1990).

## 7 GEOGRAPHIC DISTRIBUTION

Potato ring rot was first observed in Germany in 1905 (Appel, 1906; Spieckermann & Kotthoff, 1914), being the first coryneform plant‐pathogenic bacterium that was reported outside North America (Davis, 1986). In 1932 the disease was reported in Norway (Jorstad, 1932) and during the first half of the 20th century in other European countries (France) (Lansade, 1942) and the European part of Russia (Belova, 1940; Olsson, 1976). In the new world, the ring rot pathogen was observed in Canada in 1931 in the province of Quebec (Baribeau, 1931), becoming widespread across the country during 1930s (Racicot, 1944). The disease was reported for the first time in the USA in 1938 (Burkholder 1938; Starr & Riedl, 1941). By 1939 the disease had been reported in 27 states and by 1948 in 45 states of the USA (Baribeau, 1948). *C*. *sepedonicus* has also been reported in Mexico (Rueda Puente et al., 2010) while Central and South America are free of the pathogen according to EPPO. The pathogen has a clearly restricted geographical distribution in the EPPO region (McNamara & Smith, 1998). The European countries where the pathogen currently presents either in widespread or transient form include Belarus, Bulgaria, Czech Republic, Estonia, Finland, Georgia, Germany, Greece, Hungary, Latvia, Lithuania, Norway, Poland, Romania, Russia, Slovakia, Spain, Sweden, Turkey, and Ukraine. In the Netherlands, France, and the UK (Scotland), which are the primary seed‐producing areas of Europe, the pathogen is either absent or transient. In Asia, the ring rot pathogen is present in China (Jansky et al., 2009), Japan, Kazakhstan, South Korea, Nepal (Baharuddin et al., 2019; Osdaghi, 2022), Pakistan (Bhutta, 2008), and Uzbekistan (EPPO, 2022). Information about the economic impact and local distribution of ring rot disease in Asia is limited. Ring rot is absent throughout the southern hemisphere, including all of South America, Oceania, as well as the countries and territories around the equator. Although the pathogen has occasionally been reported in African countries, that is, Algeria and Egypt (Seleim et al., 2014), EPPO currently considers the pathogen to be absent from Africa. Figure S3 shows the global distribution of the ring rot pathogen as inferred by June 2021 (adapted from https://gd.eppo.int/taxon/CORBSE/distribution).

## 8 BIOLOGY AND EPIDEMIOLOGY OF THE PATHOGEN

**FIGURE 3:**
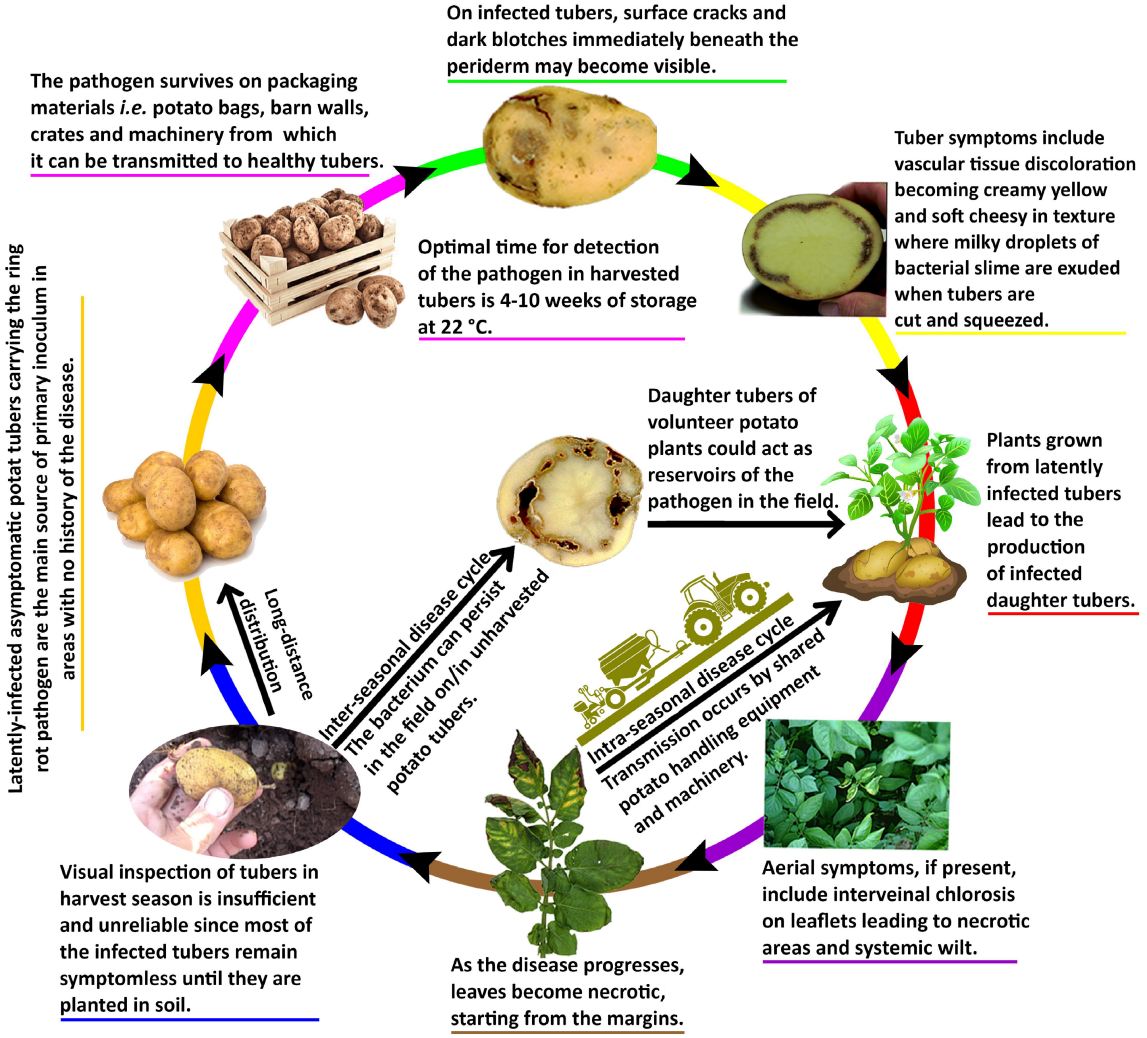
Disease cycle of bacterial ring rot of potato caused by *Clavibacter sepedonicus*

Latently infected, symptomless potato tubers carrying the ring rot pathogen are the main source of primary inoculum in areas with no history of the disease (Zielke & Naumann, 1990). The bacterium can persist in a field on the surface or inside unharvested potato tubers. Daughter tubers of volunteer potato plants growing within a nonpotato crop cultivated in rotation with potatoes could also act as reservoirs of the pathogen in the field (Pánková et al., 2007). While other species of *Clavibacter* can grow in a variety of environmental and plant‐associated niches, *C*. *sepedonicus* is almost entirely restricted to the vascular system of its host plant. Once an infected potato tuber is planted, the pathogen multiplies rapidly and passes along the vascular strands into the stems and petioles, from where it reaches the roots and maturing daughter tubers, sometimes within 8 weeks after planting. Tuber infection occurs through the stolon. Earliest infections can be observed when the tuber is cut across the heel end (Figure 2, as narrow glassy to cream‐yellow zones along the vascular tissue near the stolon end (EPPO, 2006). The pathogen is adapted to an endophytic lifestyle, proliferating within plant tissues and unable to persist in the absence of plant material (Bentley et al., 2008). A reduction in the capabilities of *C. sepedonicus* in utilizing nutrients is consistent with the fact that the environmental conditions and carbohydrate supply of the vascular system are expected to be less variable than those experienced by plant‐epiphytic or soil‐inhabiting bacteria (Bentley et al., 2008). However, there is still a high risk for the infection of healthy tubers by direct contact with superficially contaminated tubers during processing on a sieve or in a potato‐washing machine (Kakau et al., 2004). The probability of infection increases with increasing degree of contamination. Under a high primary inoculum level, 51%–93% of potato stems could be infected at 80 days after planting, leading to 10%–59% of daughter tubers being infected at harvest (De Boer & Hall, 1996). Severity of foliar and tuber symptoms is positively correlated with inoculum concentration (Westra & Slack, 1994). Cultivar and inoculum concentration interactions also impact symptom development. Figure 3 illustrates the disease cycle of bacterial ring rot in natural conditions.

*C. sepedonicus* does not survive in unsterilized field soils for long periods (van der Wolf et al., 2005), while it survives for over 29 years in sterilized soil (Ward et al., 2001). In the absence of undecomposed potato debris, it has been shown that the pathogen can survive in the soil for at least 1 year at temperatures lower than 4°C, but only for weeks at temperatures higher than 15°C (Howard et al., 1994; van der Wolf et al., 2005). The probability of infections of potatoes from infected soil by this nonmotile pathogen seems low according to experiments conducted in the USA during the 1940s (summarized by van der Wolf et al., 2005). As for free water, *C. sepedonicus* survives up to 7 days in nonsterile surface water at 10°C, while survival up to 35 days was found in sterile tap water at 20°C (van der Wolf & van Beckhoven, 2004). Rapid decline of *C. sepedonicus* cells in surface water indicates that the risk of infection of a potato crop by irrigation is low, except for cases where the contamination source is in the water, for example, symptomatic plant material that constantly releases a high population of the pathogen into the water. Wounds are necessary for entry of the ring rot bacteria into tubers, hence potato seed cutting operations enhance the spread of the pathogen (van der Wolf et al., 2005). The pathogen survives on packaging materials (potato bags, barn walls, crates, and machinery) from which it can be transferred to healthy tubers (van der Wolf et al., 2005). The pathogen remains infectious at and above freezing temperatures for at least 18 months on burlap. In another study, *C. sepedonicus* survived for 24 months on contaminated surfaces of burlap, kraft paper, and polyethylene plastic held at 12% relative humidity (RH) at either 5 or 20°C, while it persisted for fewer than 14 months on surfaces held at 94% RH at either temperature (Nelson, 1980). Storage of healthy seed tubers in contaminated crates produces infected plants (Abdel‐Kader et al., 2004).

Relative humidity, temperature, and soil profile have a significant effect on disease progress in potato plants (Pietraszko et al., 2018). *C. sepedonicus* has a low optimum growth temperature (21–23°C) and is confined mainly to cooler potato‐growing regions. Climatic conditions in north and central Europe, the northern USA, and Canada appear to favour the disease. High temperatures stimulate disease development while lower temperatures are favourable for survival of the pathogen. Symptom expression under field conditions is affected by inoculum concentration, cultivar, geographic location, and the interactions of these factors (Westra & Slack, 1994). Symptom expression in greenhouse experiments occurred faster at 22–35°C than at 16–18°C or 4°C (Eddins, 1939; Logsdon, 1967; Manzer et al., 1987). Furthermore, relatively high soil temperature favours disease development. Plant‐to‐plant transmission may occur in subsurface root systems but at very low frequency and is unlikely to play a significant role compared with the potential of transmission by shared potato‐handling equipment (Mansfeld‐Giese, 1997). Surface water is unlikely to play a role in the disease cycle. Natural dispersal is limited to infection of the maturing daughter tubers. Following establishment, the pathogen naturally spreads in the area by overwintering and spread of daughter tubers or plant materials (EFSA et al., 2019). The role of weeds and crops grown in rotation with potato in the epidemiology of the disease is yet to be determined. Furthermore, epiphytic growth of *C. sepedonicus* on nonhost plant species has a considerable role in the survival of the pathogen, as detailed above in the section 4.

## 9 GENOMIC FEATURES OF *C*. *SEPEDONICUS*

**FIGURE 4:**
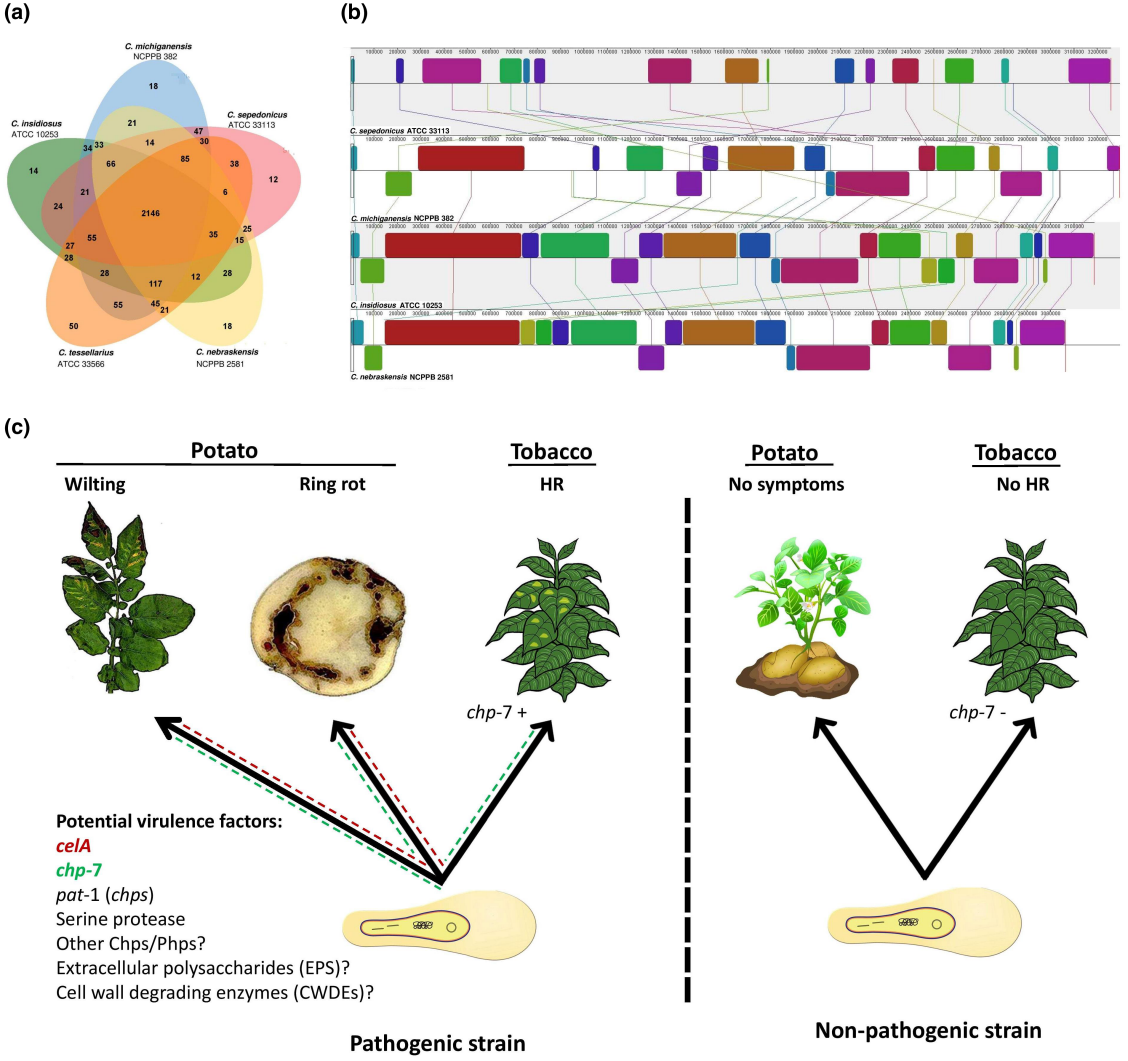
Venn diagram constructed using the OrthoVenn online service (Wang et al., 2015) showing the distribution of shared gene families (orthologous clusters) among different *Clavibacter* species (a). Pairwise alignment among the chromosomal DNA of *Clavibacter sepedonicus* ATCC 33113^T^ and three closely related *Clavibacter* species using MAUVE software (Darling et al., 2010) using default parameters (match seed weight = 15). Colours show conserved and highly related genomic regions (locally collinear blocks). Blocks shifted below the centre line indicate segments that align in the reverse orientation as inversions relative to reference strain ATCC 33113^T^. Each commonly coloured region is a locally collinear block, which is a region without rearrangement of the homologous backbone sequence. Lines between two genomes trace each orthologous locally collinear block (b). Phenotypic and genotypic differences between the pathogenic and nonpathogenic strains of *C*. *sepedonicus* on potato plants and nonhost (tobacco) plants (c)

The complete genome sequence of the type strain of *C. sepedonicus* ATCC 33113^T^ = CFBP 2049^T^ = ICMP 2535^T^ = LMG 2889^T^ = NCPPB 2137^T^ (GenBank accession number NC_010407.1) became available at the same time as that of *C. michiganensis* NCPPB 382 (Bentley et al., 2008; Gartemann et al., 2008). The complete genome resources should have entered the ring rot pathogen into the genomics era. However, only a few steps have been taken during the past decade to shed light on the genomic features and pathogenicity determinants of the species. By September 2021, only a couple of additional whole‐genome sequences had become available in the public databases, including the strains CFIA‐Cs3N and CFIA‐CsR14 with accession numbers MZMM00000000.1 and MZMN00000000.1, respectively. Whole‐genome sequence‐based phylogenomics analysis showed that *C*. *sepedonicus* strains form a monophyletic cluster phylogenetically closely related to the tomato pathogen *C. michiganensis*, still being separated from all *Clavibacter* species by average nucleotide identity (ANI) values <93% (Jacques et al., 2012; Osdaghi et al., 2020a). The chromosomal DNAs of *C. michiganensis* NCPPB 382 and *C. sepedonicus* ATCC 33113^T^ possess significant similarity while there are clear differences in their plasmid composition (Bentley et al., 2008; Eichenlaub & Gartemann, 2011). *C*. *sepedonicus* and *C. michiganensis* each have a large number of species‐specific coding sequences (CDSs, 12%–16% of all CDSs), suggesting that there may have been significant differential gene acquisition or loss since divergence from the common ancestor (Figure 4a,b). The *C. michiganensis* genome contains a large pathogenicity island (PAI, c.129 kb) known as the *chp*/*tom* region, which encodes virulence determinants while the *C. sepedonicus* genome does not contain an equivalent single large island, although it does share much of the gene content. For instance, the *C. sepedonicus* island CmsPI has significant synteny with the *tom* region of *C. michiganensis*. Furthermore, the *pat*‐*1* homologous genes in *C. michiganensis* are located exclusively in the *chp*/*tom* region, while in *C. sepedonicus* they are scattered throughout the chromosome (Bentley et al., 2008). Genomic features of *C. sepedonicus* suggest a recent adaptation for life in a restricted niche in vascular tissues of the host plant where nutrient diversity and perhaps competition are low. Comparative genomics revealed that the genome of *C. sepedonicus* has undergone recent dramatic evolution including recombination and genome rearrangements (Syverson, 2011).

The genome of *C. sepedonicus* ATCC 33113^T^ contains 106 IS elements, as evidence for extensive rearrangement of the chromosome compared to that of *C. michiganensis*. The majority of the IS elements are located in nonprotein‐encoding DNA, and only five are inserted directly into CDSs, where they are likely to have caused loss of function (Bentley et al., 2008). Similarly, the sugarcane pathogen *L. xyli* subsp. *xyli* also contains large numbers of IS elements that appear to have generated extensive genomic rearrangements, while the rarity of IS elements in the chromosome of *C. michiganensis* suggests that the IS expansion in *C. sepedonicus* was specific to this species and occurred independently and after divergence of these species. Adaptation to the niche of the vascular system would have allowed disruption of genes whose products are no longer required. The coding capacity of the *C. sepedonicus* genome is reduced due to the presence of pseudogenes comprising 3.4% of the predicted coding sequences in ATCC 33113^T^. Bentley et al. (2008) noted that most of the pseudogenes detected on the chromosome of ATCC 33113^T^ are associated with nonsense mutation, frameshift mutation, and partial deletion, while only five are due to IS insertion. Disruption of gene function in *C. sepedonicus* extends far beyond that of the identified pseudogenes. In their intact status, the pseudogenes could have encoded enzymes likely to affect the ability of *C. sepedonicus* to degrade and utilize carbohydrates, that is, cellulose, glycerol, and *N*‐acetylglucosamine. These pseudogenes are intact and functional in *C. michiganensis*, enabling it to multiply on a variety of plant surfaces. Because of this genome decay, as it stands, *C. sepedonicus* has a reduced ability to exploit previously occupied complex niches outside the plant (Bentley et al., 2008; Syverson, 2011). Furthermore, genome sequence‐based reconstruction of the metabolic networks and subsystems showed that *C. sepedonicus* ATCC 33113^T^ possesses the highest number of subsystems among *Clavibacter* species (Osdaghi et al., 2020a).

## 10 PATHOGENICITY DETERMINANTS IN THE *C. SEPEDONICUS* GENOME

Most of the gram‐negative plant‐pathogenic bacteria, that is, xanthomonads, pseudomonads, and enterobacteria, translocate a cocktail of different effector proteins (referred to as type III effectors) into host plant cells using the type III secretion system (Osdaghi et al., 2021; Shah et al., 2021). However, the type III secretion system is absent from gram‐positive plant‐pathogenic bacteria, leaving the virulence and pathogenicity mechanisms to lytic enzymes, toxic compounds, and functions on extrachromosomal plasmids (Chen et al., 2021; Eichenlaub & Gartemann, 2011; Francis et al., 2010; Hogenhout & Loria, 2008; Thapa et al., 2019). The major candidate contributors to *Clavibacter* spp. pathogenicity are EPS and enzymatic activities such as endocellulase, xylanase, polygalacturonase, and serine protease (Bentley et al., 2008; Stevens et al., 2021a; Thapa et al., 2017, 2019). The prerequisite for understanding the virulence repertories of actinobacterial plant pathogens is the development of knockout mutants and expression variants (Stevens et al., 2021a). Progress on determining the role of such putative virulence factors in *C. sepedonicus–*host interactions began advancing once it was possible to genetically manipulate the pathogen. Transposon mutagenesis and stable transformation protocols, for example plasmid vectors required for functional genetic analysis, have been developed for the ring rot pathogen (Laine et al., 1996; Nissinen et al., 2009; Syverson, 2011; Syverson & Ishimaru, 2010). Transformation efficiencies of *C. sepedonicus* are relatively low, even after optimization with vectors containing origin of replications from *Clavibacter* plasmids. Transposon mutagenesis based on Tn*1409Cβ* is prone to insertional bias into lower G + C sequences and IS elements (Laine et al., 1996; Nissinen et al., 2009). The transposase system EzTn*5* KAN‐2 created random mutations in *C. sepedonicus* and has potential for future investigations (Syverson, 2011). Functional genomics based on gene replacement of a wild‐type gene with a cloned mutated gene via homologous recombination has also been used to explore the molecular biology of *C. sepedonicus* (Lu et al., 2015; Syverson, 2011). Nevertheless, the functional genomics of *C. sepedonicus* has lagged behind that of *C. michiganensis* (Osdaghi et al., 2018d; Thapa et al., 2019). Most of the information on the virulence repertories of *Clavibacter* members are gathered from the mutagenesis, transformation, and comparative genomic analyses of the latter species (Thapa et al., 2017). *C. michiganensis* carries effectors on a c.129 kb *chp*/*tom* PAI as well as two plasmids, pCM1 and pCM2 (Meletzus et al., 1993). Fragments and/or homologs of PAI members can be found in the genomes of other pathogenic *Clavibacter* species (Osdaghi et al., 2020a). Pathogenicity and virulence of *C. sepedonicus* are probably attributed to a number of chromosomal genes, as well as genes present on extrachromosomal elements. Many strains of *C. sepedonicus* contain a circular plasmid pCS1 as an autonomously replicating plasmid or integrated into the chromosome (Mogen & Oleson, 1987). *C*. *sepedonicus* also contains one or two linear plasmids pCSL1 (90 kb) or pCSL2a (140 kb), the size of which is strain‐dependent (Bentley et al., 2008; Brown et al., 2002b). Two proteins have been demonstrated as important in the pathogenesis of *C. sepedonicus*, one of which, cellulase A, affects the severity of symptoms in potato. Cellulase A is produced by the *celA* gene that encodes an extracellular endo‐β‐1,4‐glucanase containing a plant expansin domain (Bentley et al., 2008; Hwang et al., 2019; Laine et al., 1996). The *C. michiganensis celA* gene is on plasmid pCM1, and in many strains of *C. sepedonicus celA* is located on pCS1 (Gudemstad et al., 2009; Mogen & Oleson, 1987). Loss of CelA, as mediated by loss or absence of pCS1 or by mutagenesis of *celA*, significantly reduces the severity of ring rot symptoms (Laine et al., 2000; Nissinen et al., 2001; Figure 4c).

A protein of known importance in the pathogenicity of *C. sepedonicus* is a putative serine protease required for virulence and elicitation of a nonhost HR. Pathogenic strains of *C. sepedonicus* induce an HR in tobacco leaves within 24–48 h postinfiltration while nonpathogenic strains fail to induce an HR (Lu et al., 2015; Nissinen et al., 1997, 2009; Figure 4d). The HR‐inducing activity was shown to be heat stable and protease sensitive. Although an HR has not been observed in potato leaves, a naturally occurring avirulent, HR‐negative strain did not multiply in potato, suggesting a possible correlation between host colonization, pathogenicity, and HR (Nissinen et al., 1997). Later studies revealed the HR‐inducing factor was encoded by a gene closely related to a pathogenicity gene (*pat‐1*) located on pCM2 in *C. michiganensis* (Dreier et al., 1997; Nissinen et al., 2009). Whole‐genome sequencing of the type strain ATCC 33113^T^ revealed that *C. sepedonicus* encodes 11 *pat*‐*1* homologs (Bentley et al., 2008; Gartemann et al., 2008). In *C. michiganensis* several *pat‐1* homologs and other virulence‐related genes are located within the large *chp*/*tom* PAI (Burger et al., 2005; Gartemann et al., 2008; Holtsmark et al., 2008). In contrast, eight chromosomal homologs of *pat‐1* (*chps*) are dispersed throughout the genome of *C. sepedonicus* ATCC 33113^T^, due probably to rearrangements at the sites of IS elements (Bentley et al., 2008). Three plasmid‐encoded homologs of *pat‐1* (*phps*) are also present in *C. sepedonicus*. The putative serine protease encoded by *chp‐7*, which is most similar to *pat*‐*1*, is required for triggering an HR in *Nicotiana tabacum* and for full virulence in potato and eggplant (Lu et al., 2015; Nissinen et al., 2009). In contrast to earlier findings, loss of Chp‐7 does not affect growth in planta, as a *chp*‐*7* mutant multiplied to the same extent as the wild type in eggplant (Nissinen et al., 2009). Chp‐7 expressed in the plant apoplast but not in the cytoplasm elicits an HR in *Nicotiana* species (Lu et al., 2015). The HR induction ability is lost when the putative catalytic serine residue at position 232 is mutated, suggesting that enzymatic activity is required. The HR‐inducing function of Chp‐7 is similar to that of ChpG from *C. michiganensis*, while its importance in pathogenicity is more like that of Pat‐1 (Lu et al., 2013, 2015). Two other genes, *php*‐*3* and *chp*‐*8*, have been evaluated for a role in disease severity and host colonization (Syverson, 2011). Loss of either *php*‐*3* or *chp*‐*8* did not affect elicitation of an HR in tobacco. Mutation of *php*‐*3* reduces disease severity in potato but does not affect the population size of the pathogen in eggplant or potato. Slight reductions in disease severity and population sizes in eggplant were associated with loss of *chp*‐*8* (Syverson, 2011).

While *C. sepedonicus* is a homogeneous taxon with low levels of genetic diversity, phenotypic and genotypic differences exist among strains. The ability to elicit host/nonhost responses and the extent of growth in potato or eggplant varies by strain (Brown et al., 2002a). Genomic fingerprint variations between the virulent and avirulent strains of *C. sepedonicus* have been observed (Brown et al., 2002a). Based on PCR assays with primers specific for *chp* and *php* sequences, the *chp* and *php* content varies among strains of *C. sepedonicus* (Syverson, 2011). There is little information on the impact natural mixtures within populations of *C. sepedonicus* might have on disease outcomes. However, co‐inoculation of eggplants with an avirulent HR‐negative/cellulase A‐proficient strain and an HR‐positive/cellulase‐deficient strain produced typical ring rot symptoms presumably by in planta complementation (Nissinen et al., 2001).

Genomic sequence comparisons with *C. michiganensis* and the alfalfa wilt pathogen *C. insidiosus* are yielding insights on additional potential virulence factors in the ring rot pathogen (Bentley et al., 2008; Gartemann et al., 2008; Lu et al., 2018). Several putative virulence genes have been identified, including those involved in iron uptake, detoxification of antimicrobial compounds and antibiotics, and production of cell wall‐degrading enzymes and EPSs (Bentley et al., 2008). During infection of potato with *C. sepedonicus*, six genes, including *celA*, *celB*, the xylanase gene, and two of the *pat*‐*1* homologs, were up‐regulated, suggesting a role in the pathogenicity process (Holtsmark et al., 2008). In *C. sepedonicus* ATCC 33113^T^ *celB* has been inactivated by a nonsense mutation and is probably nonfunctional (Bentley et al., 2008; Hwang et al., 2019). Genes for EPS biosynthesis are affected by genome decay in *C. sepedonicus*. Four clusters of genes required for EPS production are present in *C. sepedonicus* ATCC 33113^T^, but due to disruption from IS elements only two, EPS3 and EPS4, are functional (Bentley et al., 2008). Further studies on variation of EPS gene clusters among strains of *C. sepedonicus* could elucidate the observed variation of sugar composition and contribution of EPS to virulence (Henningson & Gudmestad, 1993; Westra & Slack, 1992). The EPS clusters in *C. michiganensis* appear to be intact and are likely to be functional. The loss of the ability to produce an EPS coat suggests that *C*. *sepedonicus* occupies a niche in which the production of such a coat is no longer advantageous or essential (Bentley et al., 2008).

Very little information is available on plant responses to *C. sepedonicus*. The target(s) of *chp*‐7 in potato or in a nonhost such as tobacco remain unknown. Infection with *C. sepedonicus* reduces transpiration and xylem function in potato plants prior to and during wilting. Transpiration depression and subsequent wilting of infected plants appears to result from reduced xylem function (Bishop & Slack, 1992). Given the similarity in virulence repertoires between *C. sepedonicus* and *C. michiganensis* and the relatedness of their hosts, it is likely that the *C. sepedonicus–*potato molecular interactions are similar to those described for *C. michiganensis–*tomato (Savidor et al., 2012; Thapa et al., 2019). However, this area of study is largely unexplored. Unlike the *C. michiganensis–*tomato model, early studies conducted in potato did not detect an effect of the plant hormone ethylene on the development of ring rot disease (Kurowski & Gudmestad, 1990).

## 11 ISOLATION, DETECTION, AND IDENTIFICATION OF THE PATHOGEN

Precultivation screening of seed potato tubers for infection with *C. sepedonicus* is the most promising approach for timely detection of the pathogen resulting in the avoidance of the pathogen before being introduced into the field. All EU members are obliged to follow the procedure in Annex I of Council Directive 93/85/EEC (EU, 1993) for detection and identification of the ring rot pathogen (EU, 1993). A detailed laboratory testing guideline in EU territory is provided by the European Food Safety Authority (EFSA et al., 2019). De Boer et al. (2017) have also provided a practical guide for detection of *C. sepedonicus* in potato tubers. The probability of accurate detection of the pathogen is positively correlated with the proportion of the infected tubers to healthy ones in a tuber lot, as well as the inoculum concentration in the infected tubers (Franc, 1999). Under field conditions, due to the ambiguity or absence of symptoms on potato plants, the results of field inspection are not sufficient to certify seed lots as being free from ring rot (EFSA et al., 2019). Field sampling of tubers should preferably be performed shortly before harvest. Furthermore, visual inspection of tubers in postharvest storage is important but insufficient for surveillance due to the fact that most of the infected tubers remain symptomless until they are planted in soil (EPPO, 2006). Semicommercial electronic devices are available to detect ring rot of potato by recognizing volatile compounds emitted by the infected potato tubers (Biondi et al., 2014). The optimal time for the detection of the pathogen in harvested daughter tubers is after 4–10 weeks of storage at 22°C (Pánková et al., 2007), while the recommended standard sample size is at least 200 tubers per 25 tonnes (EFSA et al., 2019). If an infection is confirmed, an extensive trace‐back of the origin and a trace‐forward of possible spread should be performed.

### 11.1 Isolation

Culture‐based conventional isolation methods are available for confirmation and purification of the ring rot pathogen. However, the bacteria grow relatively slowly on artificial media and are easily outcompeted by other microorganisms commonly found in environmental samples (Ward et al., 2001). Besides the basic general culture media recommended for the isolation and identification of actinobacterial plant pathogens (nutrient broth‐yeast extract [NBY], yeast extract‐peptone‐glucose agar [YPGA], and yeast extract‐dextrose‐calcium carbonate [YDC]; EPPO, 2006; Schaad et al., 2001), semiselective media are also available for the ring rot pathogen (de la Cruz et al., 1992). The semiselective media YGM (yeast extract‐glucose‐mineral salts medium supplemented with nalidixic acid and polymyxin B sulphate) and mannitol‐trimethoprim‐nalidixic acid (MTNA), either alone or in combination with an immunofluorescence colony‐staining procedure, are suitable for detection of the pathogen in naturally infected symptomless potato tubers (Jansing & Rudolph, 1998; Roozen & Van Vuurde, 1991).

### 11.2 Detection

To reliably detect the ring rot pathogen in symptomless potato tubers on a commercial scale, application of serological techniques supplemented with molecular approaches, that is, specific PCR, DNA fingerprinting, and nucleotide sequencing, is highly recommended (EFSA et al., 2019). The prerequisite for obtaining reliable results from all these techniques is an efficient standardized DNA extraction method. Several adjustments and modifications in the bacterial enrichment and DNA extraction procedure have been described (Niepold, 1999; Vreeburg et al., 2020). Martin and Beaumanoir (2001) proposed an incubation/shaking method using an extract of 200 cores of potato tubers divided into three subsamples as the most satisfactory method to detect the pathogen in potato tubers. Subsamples are diluted and analysed using immunofluorescence cell‐staining. Interestingly, the same potato heel end extract that is prepared to be used for ring rot test can also be used for detection of the other bacterial pathogens of potato, that is, soft rot pectobacteria and the brown rot pathogen *R*. *solanacearum*. Culture‐based enrichment of the bacterial population followed by specific PCR or TaqMan, a technique known as (TaqMan) BIO‐PCR, was found to be highly sensitive for screening of potato tuber extracts for *C. sepedonicus* (Kaemmerer, 2009; Schaad et al., 1999).

Serological tests such as enzyme‐linked immunosorbent assay (ELISA) were developed for detecting the ring rot pathogen in symptomless tubers (Zielke & Kalinina, 1988). Indirect ELISA was shown to be well suited for screening large numbers of samples and routine indexing of seed potatoes having consensus results among different laboratories (De Boer et al., 1992a). The ELISA results do not differ significantly among brands of ELISA plates or between laboratories (De Boer et al., 1996; De Boer & Hall, 2000a, 2000b). The populations of saprophytic bacteria also do not interfere with ELISA‐based detection of *C. sepedonicus* (Dinesen & De Boer, 1995). There is a dose–response relationship between ELISA results and bacterial concentration in plant samples (De Boer et al., 1992b).

By the beginning of the current century, several PCR‐based protocols had been developed for detection of the pathogen, including plasmidless and nonmucoid strains (Li & De Boer, 1995; Mills et al., 1997). In some cases, PCR‐based methods were shown to be more sensitive than ELISA and can be used to detect *C. sepedonicus* in symptomless potato tubers (Lee et al., 2001). Table 1 summarizes primer sets used in various DNA amplification‐based techniques for the detection and identification of the ring rot pathogen. Conventional PCR primers CMS‐6/CMS‐7, capable of amplifying a 258 bp DNA fragment from the *C. sepedonicus* plasmid pCS1, was the first PCR‐based protocol for detection of the pathogen (Schneider et al., 1993). Competitive PCR of amplicons produced by primers CMS‐6/CMS‐7 and by plant‐specific primers introduced a quantitative assay for *C. sepedonicus* along with the ability to detect false negatives (Hu et al., 1995). Three specific assays were designed using sequences of DNA fragments selected via subtraction hybridization using driver DNA of *C. insidiosus* and *C*. *michiganensis* (Mills et al., 1997). The primer sets Cms50 and Cms72 were used in an enzyme‐linked oligonucleosorbent assay in which the amplicons were hybridized in a microtitre plate with a digoxygenin‐labelled DNA probe, allowing detection of three cells in 10 µl of reaction product (Baer et al., 2001). The assay was highly specific and sensitive for detection of naturally infected tuber samples. Nested PCR with primer pair CMSIF1/CMSIR1, designed using sequences of 16S rDNA and the insertion element IS*1121*, followed by primer pair CMSIF2/CMSIR2 was 1000‐fold more sensitive for detection of *C. sepedonicus* in potato extracts than a direct PCR (Lee et al., 1997). Multiplexing the PCR with co‐amplification of the host DNA using PSA‐1/PSA‐R primers for the pathogen and NS‐7‐F/NS‐8‐R for potato enabled recognition of false‐negative PCR results (Pastrik, 2000). High‐throughput TaqMan real‐time PCR reduces costs per sample over the more labour‐intensive classical PCR (Schaad et al., 1999) and is a good addition to the detection protocols as laid down in the EU regulations, for example EU Council Directives 2006/56/EC and 2006/63/EC (Vreeburg et al., 2016). Recently, Van Vaerenbergh et al. (2017) proposed including TaqMan real‐time PCR as a primary screening test in EU/EPPO standard methods. A real‐time PCR assay based on the *celA* gene sequence is more sensitive in detecting symptomless infections of *C. sepedonicus* in seed tubers prior to planting compared to conventional PCR based on the Cms50 and Cms72a primer sets, immunofluorescence, and ELISAs (Gudmestad et al., 2009). Furthermore, addition of a reaction control to TaqMan PCR (a DNA fragment unrelated to *C. sepedonicus* flanked by the primer sequences cloned into plasmid pCmsC4) will help to validate the results, facilitating the use of TaqMan real‐time PCR in the routine testing samples for *C*. *sepedonicus* (Smith et al., 2008). More recently, on‐site detection and screening became possible by the development of the loop‐mediated isothermal amplification (LAMP) assay for detection of either all plant‐pathogenic members of *Clavibacter* as a whole (Dobhal et al., 2019) or the ring rot infection on potato (Sagcan & Kara, 2019). An AmpliDet RNA was developed for fast and specific detection of viable cells of the pathogen in complex substrates. AmpliDet RNA enables detection of 10,000 molecules of purified rRNA per reaction and 100 cfu of *C. sepedonicus* per reaction (van Beckhoven et al., 2002).

**TABLE 1:** Primer pairs used for detection and identification of *Clavibacter sepedonicus*, the causal agent of bacterial ring rot of potato

Primer name	Sequence (5′–3′)	Size of amplicon (bp)	Annealing temperature (°C)	Target	Reference
CMR16F1	GTGATGTCAGAGCTTCCTCTGGCGGAT	1425	62	*Clavibacter* spp.	Lee et al. (1997)
CMR16R1	GTACGGCTACCTTGTTACGACTTAGT				
CMS‐6	CGCTCTCCCTCACCAGACTC	258	63	*C. sepedonicus*	Schneider et al. (1993)
CMS‐7	TCCCGTGCTTGCCTGCGTTG				
CMS50F	GAGCGCGATAGAAGAGG	192	57	*C. sepedonicus*	Mills et al. (1997), Gudmestad et al. (2009)
CMS50R	TCCTGAGCAACGACAAGAAAA				
Cms50 (probe)	[DFAM] TGAAGATGCGACATGGCTCCTCGGT [DBH1]				
CMS72F	AGTTCGAGTTGATAGCAATCC	161	56	*C. sepedonicus*	Mills et al. (1997)
CMS72R	TCTCGGATTCACGATCACC				
CMS85F	AAGATCAGAAGCGACCCGCC	205	58	*C. sepedonicus*	Mills et al. (1997)
CMS85R	TCGCACAGCCAAATCCAGC				
CMSIF1^a^	TGTACTCGGCCATGACGTTGG	1066	60	*C. sepedonicus*	Lee et al. (1997)
CMSIR1^a^	TACTGGGTCATGACGTTGGT				
CMSIF2^a^	TCCCACGGTAATGCTCGTCTG	885	61	*C. sepedonicus*	Lee et al. (1997)
CMSIR2^a^	GATGAAGGGGTCAAGCTGGTC				
PSA‐1^b^	CTCCTTGTGGGGTGGGAAAA	503	58	*C. sepedonicus*	Pastrik and Rainey (1999)
PSA‐R^b^	TACTGAGATGTTTCACTTCCCC				
NS‐7‐F	GAGGCAATAACAGGTCTGTGATGC	374	62	*C. sepedonicus*	Pastrik (2000)
NS‐8‐R	TCCGCAGGTTCACCTACGGA				
Cms50‐2F	CGGAGCGCGATAGAAGAGGA	152	62	*C. sepedonicus*	Schaad et al. (1999)
Cms133R	GGCAGAGCATCGCTCAGTACC				
Cms50‐53T: TaqMan probe	AAGGAAGTCGTCGGATGAAGATGCG				
Cms72aF	CTACTTTCGCGGTAAGCAGTT	213	58		Gudmestad et al. (2009)
Cms72aR	GCAAGAATTTCGCTGCTATCC				
Cms72a (probe)	[DCY5] GATCGTGAATCCGAGACACGGTGACC [DBH2]				
Sp1F	CCTTGTGGGGTGGGAAAA	215	62	*C. sepedonicus*	Li and De Boer (1995)
Sp5r	TGTGATCCACCGGGTAAA				
CelA‐F	TCTCTCAGTCATTGTAAGATGAT	150	54		Gudmestad et al. (2009)
CelA‐R	ATTCGACCGCTCTCAAA				
CelA (probe)	[DHEX] TTCGGGCTTCAGGAGTGCGTGT [DBH2]				
Inner primer: CM‐FIP (LAMP)	TCTGAGTCGGACGCGCTCCGTGTGGCGGAGGAGGAA	NA	65	*Clavibacter* spp.	Dobhal et al. (2019)
Inner primer: CM‐BIP (LAMP)	CAAAGCGCCCCTCCAGCTTCTACGGGTTCATCGCCCTC	NA	65		
Outer primer: CM‐F3 (LAMP)	ACCGTCTCCTTGATGGAGTG	NA	65		
Outer primer: CM‐B3 (LAMP)	GCCGAACCTCTGGGTGT	NA	65		
internal loop primer: CM‐LF (LAMP)	CGCATCATCGTCGAGAACGT	NA	65		
internal loop primer: CM‐LB (LAMP)	CAGGAGGCTCAGGAGCGAGA	NA	65		
Inner primer: FIP (F1c‐F2) (LAMP)	GCGGACATTCAAGGACCGAGG‐CGTGATCAAGGAAGTCGTCG	NA	70		Sagcan and Kara (2019)
Inner primer: BIP (B1c‐B2) (LAMP)	CAGGTCACCACGGTACTGAGC‐GTCCTGAGCAACGACAAGA	NA	70		
Outer primer: F3 (LAMP)	GCGCGATAGAAGAGGAACTC	NA	70		
Outer primer: B3 (LAMP)	GGACATCTCTCAGGTGCCA	NA	70		
Probe (LAMP)	FAM‐GGCTTTTGCCAGATT	NA	70		

Abbreviation: LAMP, loop‐mediated isothermal amplification.

^a^ To be used in nested PCR with primer pair CMSIF1/CMSIR1 followed by primer pair CMSIF2/CMSIR2 (Lee et al., 1997).

^b^ This primer pair could be used either alone (Pastrik & Rainey, 1999) or in a multiplex PCR with NS‐7‐F/NS‐8‐R as an internal PCR control (Pastrik, 2000).

Due to the equivalent economic importance of the other bacterial pathogens in potato, that is, soft rot agents of the genera *Pectobacterium* and *Dickeya* as well as *R. solanacearum*, simultaneous detection of all these pathogens in a single sample has always been a matter of interest to reduce the cost and effort required in quarantine surveys (Nikitin et al., 2018). Test performance comparisons among 10 official testing laboratories highlighted the importance of appropriate pathogen DNA extraction protocols (Vreeburg et al., 2020). A multiplex real‐time PCR assay was developed by Massart et al. (2014) for simultaneous detection of *R. solanacearum* race 3 and *C. sepedonicus* in potato tubers. Real‐time PCR tests and immunofluorescence proved to be sensitive and specific for simultaneous detection of *C. sepedonicus* and *R. solanacearum* in potato tubers (Vreeburg et al., 2016). Molecular diagnostic probes (Firrao, 1990) and genome‐wide diagnostic microarray systems (Aittamaa et al., 2008) have been developed for simultaneous detection and identification of different bacterial pathogens of potato, including *C. sepedonicus* (Arahal et al., 2004; Degefu et al., 2016). Validation of four TaqMan real‐time PCRs for the detection of *R. solanacearum*, *R. pseudosolanacearum*, and *C*. *sepedonicus* in potato tubers using a statistical regression approach has recently been performed (Vreeburg et al., 2018). Hence, since 2017, TaqMan real‐time PCR has been recommended for inclusion in EU Directives and EPPO standards as a reliable primary screening method along with the existing screening tests, that is, immunofluorescence, conventional PCR, semiselective plating, and bioassay (Van Vaerenbergh et al., 2017).

### 11.3 Identification

When typical ring rot symptoms and oozing are observed on potato tubers, the disease can readily be confirmed by direct application of gram‐staining or/and a serological test of the tuber exudates, in which a positive result is the presence of gram‐positive bacterial cells or specific antigen, respectively (Manzer & Slack, 1979). The colony colour and pigmentation on solid media, for example YPGA, NBY agar, and YDC, is a useful diagnostic characteristic for coryneform plant‐pathogenic bacteria (Davis, 1986; Hamidizade et al., 2020; Osdaghi et al., 2020b; Vidaver, 1982). Despite all the other plant‐pathogenic coryneform bacteria, *C*. *sepedonicus* is usually nonpigmented on solid media (Carlson & Vidaver, 1982). Due to the high level of homogeneity within *C. sepedonicus* strains, API 50CH and API ZYM systems were reliably applied for identification of the pathogen (Palomo et al., 2006). Furthermore, repetitive sequence‐derived PCR (rep‐PCR)‐based genomic fingerprinting as well as two primers randomly amplified the polymorphic DNA (TP‐RAPD) technique to rapidly and reliably differentiate *C. sepedonicus* from other *Clavibacter* species (Louws et al., 1998; Rivas et al., 2002; Smith et al., 2001). The use of matrix‐assisted laser desorption ionization‐time of flight mass spectrometry (MALDI‐TOF MS) was shown to be an effective method for identification of *C. sepedonicus*. All the *Clavibacter* species generate distinct and reproducible MALDI‐TOF MS profiles, with unique and specific ion peaks as biomarkers for identification (Zaluga et al., 2011). Nucleotide sequence‐based phylogenetic analyses using either a single housekeeping gene sequence (e.g., *gyrB*; Zaluga et al., 2011) or multilocus sequence analysis (MLSA) of concatenated sequences of different genes (Jacques et al., 2012) reliably identify species of *Clavibacter*, including *C. sepedonicus*. Amplification and sequencing of the *gyrB* gene using a single primer set has sufficient resolution and specificity to identify each species within the genus (Ansari et al., 2019; Osdaghi et al., 2018a; Zaluga et al., 2011).

## 12 MANAGEMENT

Management of ring rot disease is mainly based on the principle of exclusion by the use of pathogen‐free tubers for planting. Screening potato tubers prior to cultivation is the most effective approach to detecting and eliminating the ring rot pathogen. The EU Control Directive for ring rot puts in place community‐wide measures for surveillance, containment, and eradication of the pathogen, while countries where the disease is absent take strong phytosanitary measures to prevent its introduction (McNamara & Smith, 1998).

Regarding pathogen exclusion strategy, strict hygiene is a key element in the management of bacterial ring rot. Given the pathogen's limited survival in field soil and surface water, and on plant debris, most hygiene measures target its relatively longer persistence on inert surfaces (van der Wolf et al., 2005). Infected packaging equipment and storage facilities, that is, potato crates, bags, vehicles, and machinery, support the survival of the pathogen in short and medium timeframes and are important in spreading the pathogen to healthy lots of seed potatoes (EFSA et al., 2019). Sodium hypochlorite is an effective disinfectant for decreasing the pathogen's survival on wooden surfaces, while hydrogen peroxide is common for treating metal surfaces of agricultural machines and other equipment (Howard et al., 2015). Prior to disinfection, surfaces should be washed because dirt negatively affects the efficacy of the disinfectant (Stevens et al., 2021b). To eradicate the pathogen on wooden potato storage crates, jet cleaning in a crate washer for 2 min using the authorized dose of sodium‐*p*‐toluenesulfochloramide has been shown to be an effective method for disinfection (Stevens et al., 2017). Phthalocyanine can be used to destroy the pathogen on the surfaces of contaminated tubers or other objects (Lewosz & Pastuszewska, 1995). Flusulfamide has a protective but not a curative effect against *C*. *sepedonicus* (Slack & Westra, 1998). Selenium nanocomposite is a new antimicrobial agent for possible plant sanitation because it has a bactericidal effect on *C. sepedonicus* and no apparent negative side effects on the host plant (Papkina et al., 2015; Perfileva et al., 2018a). As for the marketability and commercial status of these agents, sodium hypochlorite, hydrogen peroxide, and sodium‐*p*‐toluenesulfochloramide are allowed to be used as cleaning agents in EU territories, but activity against plant pathogens cannot be claimed, and the products need to be registered as plant protection agents, which is an expensive and long‐term process. The only biocide registered as a plant protection agent is Menno Florades (Baysal‐Gurel et al., 2013), which is, however, less effective than sodium‐*p*‐toluenesulfochloramide. Flusulfamide is used as a fungicide to control clubroot of brassicas caused by *Plasmodiophora brassicae*, but it is banned in the EU. Selenium nanocomposite particles are not registered as a plant protection agent, but they are interesting because they also have a biomedical potential to boost the immune response of humans. In the USA, some products based on organic materials and essential oils are on the market (e.g., EcoTrol), but these products need further testing for ring rot control in potato (Miller et al., 2008). Phthalocyanine is a low‐toxicity, broad‐spectrum bactericide, but has been used only as an experimental agent and is not used in practice. No biocontrol agent is registered as a plant protection agent for control of ring rot in potato in the EU. During disposal of infected ware or seed lots, tubers should be treated (by heat or chemicals) or processed so that there is no risk of the organism surviving or there is no risk of escape from a waste disposal site to agricultural land. No potatoes should be grown on contaminated land for at least 3–4 years, during which time volunteer potato plants should also be eliminated.

Management strategies aimed at chemical or biological protection have been explored but probably have limited potential for use in seed potato production because of concerns of latent infections and the potential for extensive pathogen spread (De Boer & Boucher, 2011). Furthermore, over‐reliance on copper‐based chemicals in agriculture has resulted in environmental and groundwater pollution (Lamichhane et al., 2018). Heat treatments of potato to eliminate *C. sepedonicus* from tubers have been investigated with mixed results (Kaemmerer, 2009). Recycling of plant materials by means of composting and subsequent application of the produced humus to the environment has become a common practice, with a risk of spreading *C. sepedonicus*. Throughout composting, during degradation of the organic substrates heat is produced, resulting in an increase in temperature up to 50–70°C (Gurtler et al., 2018). Viable pathogen cells were extracted after composting potato tubers and debris for 6 days at 70°C, 13 days at 55°C, and a 90 min pasteurization at 70°C, indicating that *C*. *sepedonicus* might be disseminated through potato residues from processing industries (Steinmöller et al., 2013). Stevens et al. (2021c) noted that the pathogen was eradicated by exposure to heat after a treatment for 60 min at 55°C. Biofilm formation by *C. sepedonicus* is suspected to play a role in disease but has not been examined methodically. In an in vitro assay, exposure to sodium monoiodoacetate as well as Lazurite preparation reduced biofilm formation (Perfileva et al., 2018b). It is notable that bacteria in a biofilm state are more resistant to chemical treatments than planktonic cells (Howard et al., 2015), and therefore disinfection of materials should be preceded by, or combined with, disruption of the biofilm matrix through washing (Stevens et al., 2017). A combination of moderate heat shock (45°C) and treatment with the glycolysis inhibitor monoiodoacetate negatively affected *C. sepedonicus* in vitro (Rymareva et al., 2008). Plant‐based antimicrobial compounds, essential oils, and volatile organic compounds produced by *Bacillus subtilis* were shown to suppress *C. sepedonicus* (Cai et al., 2014, 2020; Rajer et al., 2017). Gamard and De Boer (1995) identified several bacterial strains antagonistic to *C. sepedonicus* in vitro; three of the strains were evaluated under field conditions and either increased plant stands or reduced disease incidence when co‐inoculated with the pathogen on potato seed (Gamard & De Boer, 1995).

## 13 HOST RESISTANCE

Potato cultivars that are completely immune or resistant to ring rot are unavailable. However, immunity was detected in the disomic tetraploid 2EBN species *Solanum acaule*, suggesting the possibility of being transferred into the cultivated potato (Kriel et al., 1995a). *S*. *acaule* possesses a temperature‐dependent immunity to infection: at 21°C it is immune to *C*. *sepedonicus* but at 15°C it supports a large population of the pathogen (Laurila et al., 2003). Hence, *S. acaule* appears to be a good source of immunity for introgression studies (Kriel et al., 1995b). Somatic hybrids between *S. acaule* and *S. tuberosum* with three different genome ratios expressed symptoms of ring rot and were susceptible to infection; the genome compositions of the hybrids influenced bacterial titre (Laurila et al., 2003). Tolerant cultivars remaining symptomless can be infected and serve as carriers of the pathogen to the next generation (De Boer & McCann, 1990).

## 14 CONCLUSION AND FUTURE AVENUES FOR RESEARCH

Bacterial ring rot disease caused by *C. sepedonicus* was once considered a devastating disease in many potato production regions. While the disease remains a very serious threat, the devastation it caused in the 1930s and 1940s has been largely attenuated by strict seed potato certification programmes with zero tolerances for the disease that have been implemented in most potato‐growing countries. During the last 20–30 years, incidence of the disease has been significantly further reduced by testing symptom‐free seed lots using serological and molecular methods designed to detect latent ring rot infections. In some regions of Canada and the EU, functional eradication has been achieved (De Boer & Boucher, 2011): the disease does not occur over a number of years and the pathogen cannot be detected by sensitive laboratory testing of seed lot samples, but total eradication cannot be proven due to the intractable nature of the pathogen.

During the past two decades, genomics has played an increasing role in the understanding of colonization, infection, transmission, and evolution of plant‐pathogenic bacteria. Comparative genomics analyses and mutagenesis have already revealed a number of pathogenicity‐related genes in *C. sepedonicus*. Improvements in available gene replacement strategies for *C. sepedonicus* are needed to enable large‐scale evaluation of the bacterial genes involved in plant pathogenesis. Sequencing of additional strains of *C. sepedonicus* and subsequent genome comparisons will provide genome‐informed improvements in detection methods to trace tuber infections with lower efforts and cost. Further studies on plant and pathogen transciptomics and metatranscriptomics will initiate a deeper understanding of the molecular basis for the endophytic lifestyle of *C. sepedonicus* in potato. Such knowledge could aid in mitigating the negative impacts of latent infections in potato production. Furthermore, transcriptomic responses of various potato varieties at different stages of bacterial infection may provide deeper insights into the molecular basis of plant susceptibility and immunity to ring rot. Such knowledge could enable breeding for durable broad‐spectrum resistance and disease control strategies that are acceptable to the potato industry. Finally, recent advances in our understanding of molecular host–pathogen interactions of other plant pathogens in the *Microbacteriaceae* will continue to aid development of a more comprehensive understanding of the molecular biology of *C. sepedonicus* and identify research paths for the sustainable management of bacterial ring rot in the 21st century.

## CONFLICT OF INTEREST

The authors declare that the research was conducted in the absence of any commercial or financial relationships that could be construed as a potential conflict of interest.

## AUTHOR CONTRIBUTIONS

E.O. conceived and designed the work. E.O., H.A., and X.L. designed and constructed the figures and graphics with assistance from J.M.v.d.W., C.A.I., and S.H.d.B. All the co‐authors contributed to writing different sections of the manuscript.

## Supporting information


**FIGURE S1** Multilocus sequence analysis using concatenated sequences of *atpD, dnaK, gyrB, ppK, recA*, and *rpoB* genes in plant‐pathogenic members of *Clavibacter*. Neighbour‐joining tree was generated based on sequences of 60 *Clavibacter* strains and rooted using *Rathayibacter iranicus* CFBP 807 as outgroup. All *C. sepedonicus* strains are clustered in a monophyletic clade phylogenetically related to the tomato pathogen *C. michiganensis*. Bootstrap values (>50%) are shown at branch points. Numbers following taxon names indicate number of strains I each clade. Adapted from Tian et al. (2021)

**FIGURE S2** Symptoms of bacterial ring rot on potato (a–c) and sugar beet (d–f) plants artificially inoculated with *Clavibacter sepedonicus* under greenhouse conditions. On potato, infected plants may be smaller in size (a; left plant is infected while the right plant is healthy). Interveinal chlorosis followed by necrotic areas are observed 10–12 days postinoculation on potato leaflets (b,c). On sugar beet, infected young petioles are curled, and whole leaves are distorted

**FIGURE S3** Geographic distribution of bacterial ring rot of potato caused by *Clavibacter sepedonicus*. The data obtained from EPPO and CABI databases up to June 2021. Green circles indicate the presence of the pathogen while blue circle shows the status of the pathogen under eradication. The source map is from https://commons.wikimedia.org/wiki/File:A_large_blank_world_map_with_oceans_marked_in_blue.PNG

## Data Availability

Data sharing is not applicable to this article as no new data were created or analysed.
