# Supplementary figures and images for: Bacterial ring rot of potato caused by Clavibacter sepedonicus: A successful example of defeating the enemy under international regulations
Source: Mol Plant Pathol. 2022 Feb 10;23(7):911–32. doi: 10.1111/mpp.13191 (PMC9190974; doi:10.1111/mpp.13191)

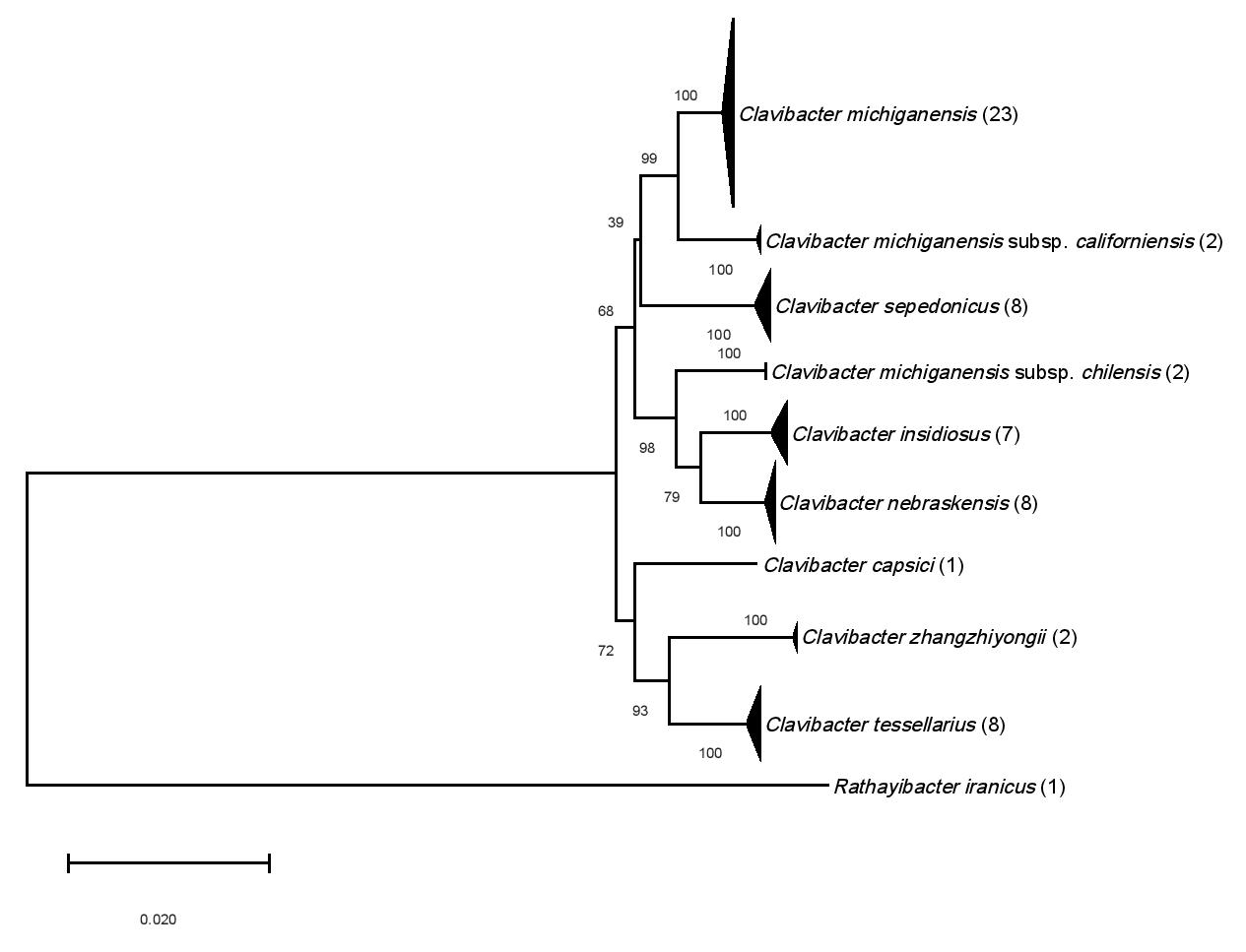

Supplement: Supplementary file 1 — FIGURE S1 Multilocus sequence analysis using concatenated sequences of atpD, dnaK, gyrB, ppK, recA, and rpoB genes in plant‐pathogenic members of Clavibacter. Neighbour‐joining tree was generated based on sequences of 60 Clavibacter strains and rooted using Rathayibacter iranicus CFBP 807 as outgroup. All C. sepedonicus strains are clustered in a monophyletic clade phylogenetically related to the tomato pathogen C. michiganensis. Bootstrap values (>50%) are shown at branch points. Numbers following taxon names indicate number of strains I each clade. Adapted from Tian et al. (2021) [file MPP-23-911-s002.jpg]

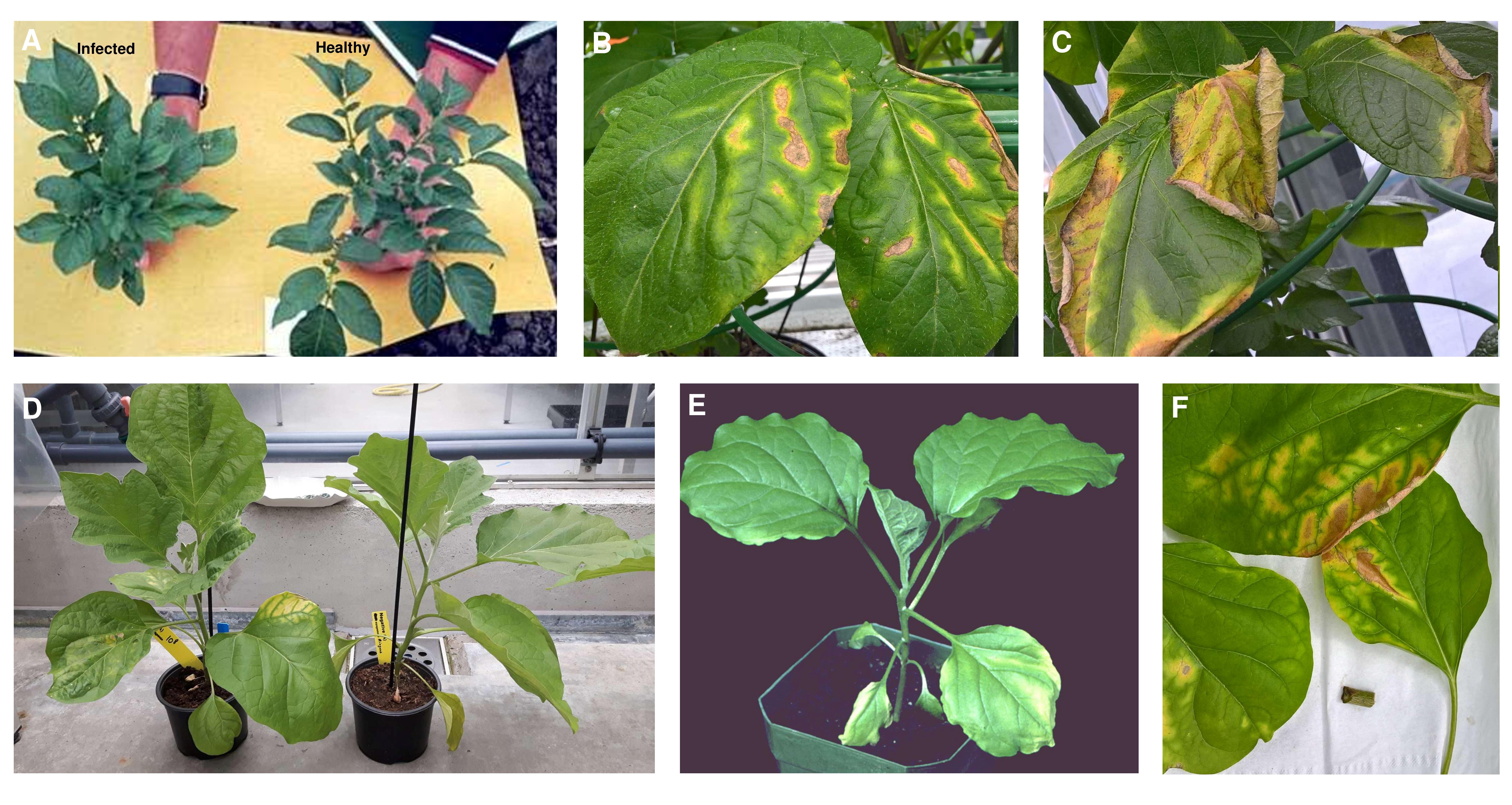

Supplement: Supplementary file 2 — FIGURE S2 Symptoms of bacterial ring rot on potato (a–c) and sugar beet (d–f) plants artificially inoculated with Clavibacter sepedonicus under greenhouse conditions. On potato, infected plants may be smaller in size (a; left plant is infected while the right plant is healthy). Interveinal chlorosis followed by necrotic areas are observed 10–12 days postinoculation on potato leaflets (b,c). On sugar beet, infected young petioles are curled, and whole leaves are distorted [file MPP-23-911-s001.jpg]

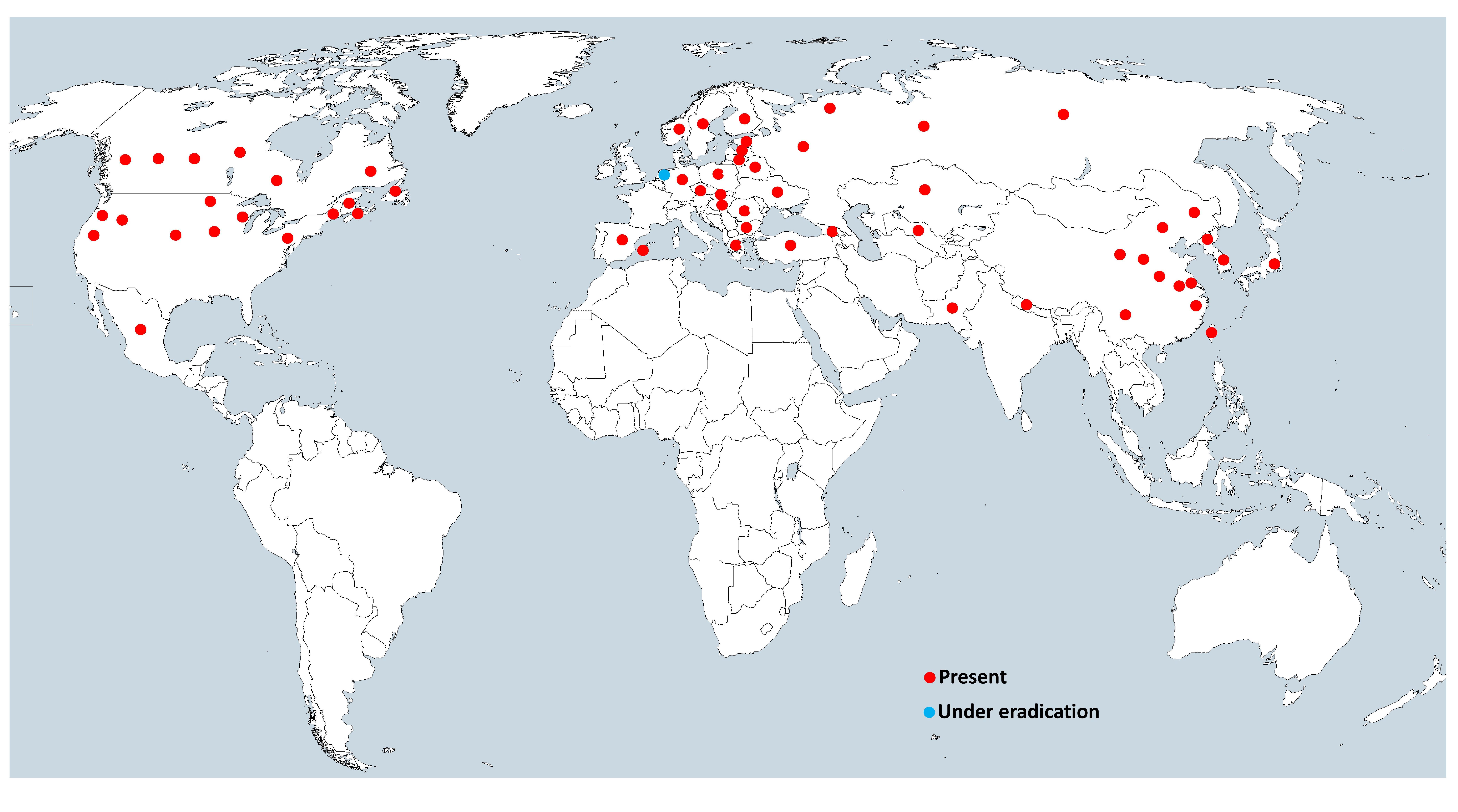

Supplement: Supplementary file 3 — FIGURE S3 Geographic distribution of bacterial ring rot of potato caused by Clavibacter sepedonicus. The data obtained from EPPO and CABI databases up to June 2021. Green circles indicate the presence of the pathogen while blue circle shows the status of the pathogen under eradication. The source map is from https://commons.wikimedia.org/wiki/File:A_large_blank_world_map_with_oceans_marked_in_blue.PNG [file MPP-23-911-s003.jpg]
